# From Tumour to Bloodstream: Advancing PD‐L1 Testing With Liquid Biopsies in Non‐Small Cell Lung Cancer

**DOI:** 10.1111/cyt.70094

**Published:** 2026-05-30

**Authors:** R. Leporati, V. Callegari, A. Ancora, M. Palmieri, B. Riccò, E. Cabitza, L. Trudu, K. Di Emidio, C. Chiavelli, A. Eccher, I. Mastrolia, V. Catani, S. Bettelli, G. Guaitoli, M. Dominici, F. Bertolini

**Affiliations:** ^1^ Division of Medical Oncology, Department of Oncology and Hematology University Hospital of Modena Modena Italy; ^2^ Department of Medical and Surgical Sciences for Children and Adults University of Modena and Reggio Emilia Modena Italy; ^3^ Clinical and Experimental Medicine PhD Program University of Modena and Reggio Emilia Modena Italy; ^4^ Laboratory of Cellular Therapy, Department of Medical and Surgical Sciences for Children and Adults University Hospital of Modena Modena Italy; ^5^ Department of Diagnostic Medicine and Public Health University of Modena and Reggio Emilia ‐ Section of Pathology Modena Italy; ^6^ Molecular Pathology and Predictive Medicine University Hospital of Modena Modena Italy

**Keywords:** CTC, ctDNA, EV, immunotherapy, liquid biopsy, lung cancer, NSCLC, PD‐L1

## Abstract

Immune checkpoint inhibitors (ICIs) have revolutionised the treatment landscape of non‐small cell lung cancer (NSCLC) without actionable genomic alterations by opening new possibilities for treatment. However, the predictive value of Programmed Death‐Ligand 1 (PD‐L1) expression assessed by immunohistochemistry (IHC) is quite modest, leaving many patients without optimal benefit from ICIs. To address this gap, liquid biopsy is gaining momentum as a promising tool to complement tissue‐based PD‐L1 assessment. Liquid biopsy, which entails the analysis of circulating tumour cells (CTCs), extracellular vesicles (EVs), circulating tumour DNA (ctDNA), and other biomarkers, offers a non‐invasive approach for tracking disease evolution and interactions with the immune system. We conducted a narrative review to explore the potential of liquid biopsy in enhancing patient selection for ICIs, its predictive and prognostic value in advanced NSCLC, and the associated technical and clinical implications. By highlighting recent advancements and ongoing research efforts, we underscore the critical role of liquid biopsy in driving precision immuno‐oncology. Establishing robust, reproducible methods for PD‐L1 assessment through liquid biopsy is essential for translating this approach into clinical practice, with the potential to overcome the limitations of tissue‐based assays and better individualise immunotherapy strategies in NSCLC.

## Graphical abstract

This review explores liquid biopsy as a complementary approach to tissue‐based PD‐L1 assessment in advanced NSCLC treated with immune checkpoint inhibitors. Focusing on circulating tumour cells, extracellular vesicles, and circulating tumour DNA, it summarises current evidence, technical challenges, and future perspectives for personalised immunotherapy.

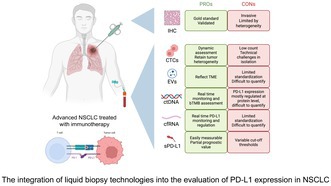

**Abbreviations**
cfRNA: cell‐free RNA
CTCs: circulating tumour cells
ctDNA: circulating tumour DNA
Evs: extracellular vesicles
IHC: immunohistochemistry
NSCLC: non‐small cell lung cancer
PD‐L1: Programmed Death‐Ligand 1
sPD‐L1: soluble PD‐L1

## 1 Introduction

Non‐small cell lung cancer (NSCLC) management has changed significantly over the last 10 years. For patients without actionable genomic alterations, immune checkpoint inhibitors (ICIs) represent the backbone of therapy [1, 2]. By restoring T‐cell activity and enhancing antitumor immune responses, ICIs targeting Programmed Death‐1 (PD‐1), its ligand Programmed Death‐Ligand 1 (PD‐L1), and Cytotoxic T‐Lymphocyte‐Associated protein 4 (CTLA‐4) have demonstrated clinically meaningful benefits across multiple disease settings. These include first‐line therapy for metastatic disease, consolidation treatment in locally advanced NSCLC, and, more recently, neoadjuvant and adjuvant strategies [3, 4, 5, 6, 7, 8, 9]. Despite these advances, important challenges remain. Primary and acquired resistance to ICIs is common, with approximately 70% of patients with advanced NSCLC failing to achieve a durable benefit. Moreover, reliable biomarkers capable of predicting immunotherapy efficacy remain limited. While several candidates, such as tumour mutational burden (TMB), have been extensively investigated, none have yet been integrated into routine clinical practice [10].

To date, PD‐L1 expression assessed by immunohistochemistry (IHC) on tumour tissue remains the only approved marker for ICIs efficacy, and international guidelines recommend its testing for treatment decision‐making in NSCLC [1, 2, 11]. Although numerous clinical trials have demonstrated the utility of PD‐L1 expression in enhancing patient selection and in optimising therapeutic outcomes, its limitations have also been widely acknowledged [3, 9, 12, 13, 14, 15, 16]. One major limitation is the inter‐ and intra‐tumour heterogeneity of PD‐L1 expression, which has been reported not only between primary and metastatictumours, but also among different regions within the sametumour. As a result, small tumour biopsies can sometimes fail to capture the full spectrum of PD‐L1 expression, leading to potential underestimation of its status [17, 18, 19, 20, 21, 22]. Moreover, collecting adequate tissue samples for PD‐L1 analysis may be challenging. In some cases, the size or location of the tumour may hinder the need for a biopsy, and cytologic smears, often the only available samples, are not approved for PD‐L1 testing unless made into cell‐block preparations through fixation [17, 20]. In addition, variability among commercially available assays, differences in antibodies, staining platforms, scoring systems, and cut‐off thresholds introduce further uncertainty and may influence treatment eligibility [14]. Taken together, these limitations underscore the growing need to explore novel approaches for PD‐L1 evaluation.

Liquid biopsy has emerged as a minimally invasive approach that enables the analysis of tumour‐derived material in peripheral blood, including circulating tumour cells (CTCs), extracellular vesicles (EVs), circulating tumour DNA (ctDNA), cell‐free RNA (cfRNA), and soluble proteins [23]. Unlike tissue biopsies, liquid biopsies offer the possibility of repeated sampling over time, potentially capturing dynamic changes in tumour biology and treatment‐induced immune modulation, particularly in settings where tissue is unavailable or insufficient, or when longitudinal monitoring is desired [23]. Considering its potential to address the limitations of PD‐L1 assessment through tissue‐based approaches, liquid biopsy is considered a key tool in refining immunotherapy decision‐making for NSCLC.

We conducted a narrative review of the literature to provide an overview of current evidence on PD‐L1 assessment using liquid biopsy approaches in advanced NSCLC, critically discussing the biological rationale, technical considerations, and emerging clinical implications of different blood‐based PD‐L1‐related biomarkers, while highlighting both their potential and their current limitations.

## 2 Methods

This review was conceived as a narrative overview of the current landscape of PD‐L1 assessment through liquid biopsy in advanced NSCLC, with the aim of discussing both biological rationale and potential clinical applications rather than performing a formal systematic review or meta‐analysis. A literature search was conducted using PubMed and Embase, focusing on studies published between January 2015 and January 2025. Search terms included combinations of keywords related to NSCLC, PD‐L1, liquid biopsy, and immunotherapy. An example search string was: (“non‐small cell lung cancer” OR NSCLC) AND (“PD‐L1” OR “programmed death‐ligand 1”) AND (“liquid biopsy” OR “circulating tumour cells” OR CTC OR “circulating tumour DNA” OR ctDNA OR “extracellular vesicles” OR EV OR “exosomes”) AND (“immunotherapy” OR “immune checkpoint inhibitors” OR ICI). Studies were considered eligible when they investigated PD‐L1 expression, PD‐L1‐associated biomarkers, or related immune signatures in blood‐derived samples from patients with NSCLC, particularly in the context of immunotherapy treatment. Preference was given to original clinical and translational studies with potential clinical relevance. However, because this remains a rapidly evolving field characterised by substantial methodological heterogeneity, selected exploratory and proof‐of‐concept studies were also included when deemed biologically informative and relevant to the aims of the review. Articles not directly aligned with the topic of interest, including editorials, narrative commentaries, isolated case reports, and conference abstracts lacking adequate methodological information, were not considered. Non‐English publications were also excluded. Study selection was based on relevance after screening titles and abstracts, followed by full‐text evaluation of potentially pertinent articles.

## 3 Current Methods for PD‐L1 Assessment in NSCLC

PD‐L1 is a type I transmembrane protein belonging to the B7 family and is expressed on a wide range of immune and non‐immune cells, including tumour cells. By engaging PD‐1 on T cells, tumour‐associated PD‐L1 contributes to immune escape and tumour progression [24, 25]. Among immune checkpoint molecules, PD‐L1 is the inhibitory membrane ligand that has been most extensively studied in NSCLC [24]. In routine practice, PD‐L1 expression is assessed using IHC on formalin‐fixed, paraffin‐embedded tumour tissue. Several companion and complementary diagnostic assays are commercially available, each developed in association with specific ICIs [26]. These include Dako 22C3, Dako 28–8, Ventana SP263, and Ventana SP142. While 22C3, 28–8, and SP263 primarily evaluate PD‐L1 expression on tumour cells, SP142 also incorporates immune cell staining, often resulting in lower tumour PD‐L1 scores. Comparative studies have shown substantial concordance among 22C3, 28–8, and SP263, whereas SP142 tends to underestimate PD‐L1 expression, particularly at higher cut‐offs [27].

In advanced NSCLC, approximately half of tumours exhibit PD‐L1 expression with a tumour proportion score (TPS) ≥ 1%, and around one‐third display high expression (TPS ≥ 50%) [26]. Reported positivity rates vary widely across studies, reflecting differences in assay selection, scoring criteria, tumour biology, and pre‐analytical variables. Inter‐assay variability, focal expression patterns, and sampling bias further limit the reproducibility of PD‐L1 IHC, reinforcing the need for complementary biomarkers [26, 28, 29]. Automated image analysis and digital pathology tools are being explored to improve scoring reproducibility and integrate PD‐L1 assessment with additional immune parameters, such as CD8+ T‐cell infiltration [26]. While promising, these approaches still require extensive clinical validation. Table 1 summarises PD‐L1 assays and cut‐offs used in phase III clinical trials on ICIs in advanced NSCLC.

**TABLE 1:** Phase III trials of ICIs in advanced NSCLC and PD‐L1 evaluation.

Clinical Trial	Reference	Treatment arms	PD‐L1 assay	PD‐L1 cut‐off	PD‐L1 positivity rate	Study population
KEYNOTE‐024	7	Pembrolizumab vs. pb‐CT	Dako, 22C3	TPS ≥ 50%	30.2%	Advanced NSCLC, 1st line
Impower‐110	8	Atezolizumab vs. pb‐CT	Ventana, SP142	TC or IC	16%	Advanced NSCLC, 1st line
EMPOWER‐Lung01	9	Cemiplimab vs. pb‐CT	Dako, 22C3	TPS ≥ 50%	28%	Advanced NSCLC, 1st line
KEYNOTE‐189	3	Pembrolizumab + pb‐CT vs. pb‐CT	Dako, 22C3	TPS ≥ 1% TPS ≥ 50%	63.7%	Advanced NSCLC, 1st line
KEYNOTE‐407	4	Pembrolizumab + pb‐CT vs. CT	Dako, 22C3	TPS ≥ 1% TPS ≥ 50%	62.1%	Advanced NSCLC, 1st line
CheckMate‐9LA	5	Nivolumab‐ Ipilimumab + pb‐CT vs. CT	Dako, 28–8	TPS ≥ 1%	60%	Advanced NSCLC, 1st line
EMPOWER‐Lung03	6	Cemiplimab + pb‐CT vs. CT	Dako, 22C3	TPS ≥ 1%	63%	Advanced NSCLC, 1st line
CheckMate‐017	14	Nivolumab vs. Docetaxel	Dako, 28–8	TPS ≥ 1%	83%	Advanced Squamous NSCLC, 2nd line
CheckMate‐057	13	Nivolumab vs. Docetaxel	Dako, 28–8	TPS ≥ 1%	78%	Advanced Non‐squamous NSCLC, 2nd line
KEYNOTE‐01	15	Pembrolizumab vs. Docetaxel	Dako, 22C3	TPS ≥ 1% TPS ≥ 50%	66% (≥ 1%) 33% (≥ 50%)	Advanced NSCLC, 2nd line
OAK	12	Atezolizumab vs. Docetaxel	Ventana, SP142	≥ 1% IC TC1/2/3 or IC1/2/3	55%	Advanced NSCLC, 2nd line
PACIFIC	16	Durvalumab vs. Placebo	Ventana, SP263	TPS ≥ 1%	63%	Unresectable stage III NSCLC

Abbreviations: CPS, Combined Positive Score; CT, Chemotherapy; IC, Immune Cells; NSCLC, Non‐Small Cell Lung Cancer; pb‐CT, platinum‐based chemotherapy; TC, Tumour Cells; TPS, Tumour Proportion Score.

## 4 Liquid Biopsy: An Emerging Complementary Tool

**FIGURE 1:**
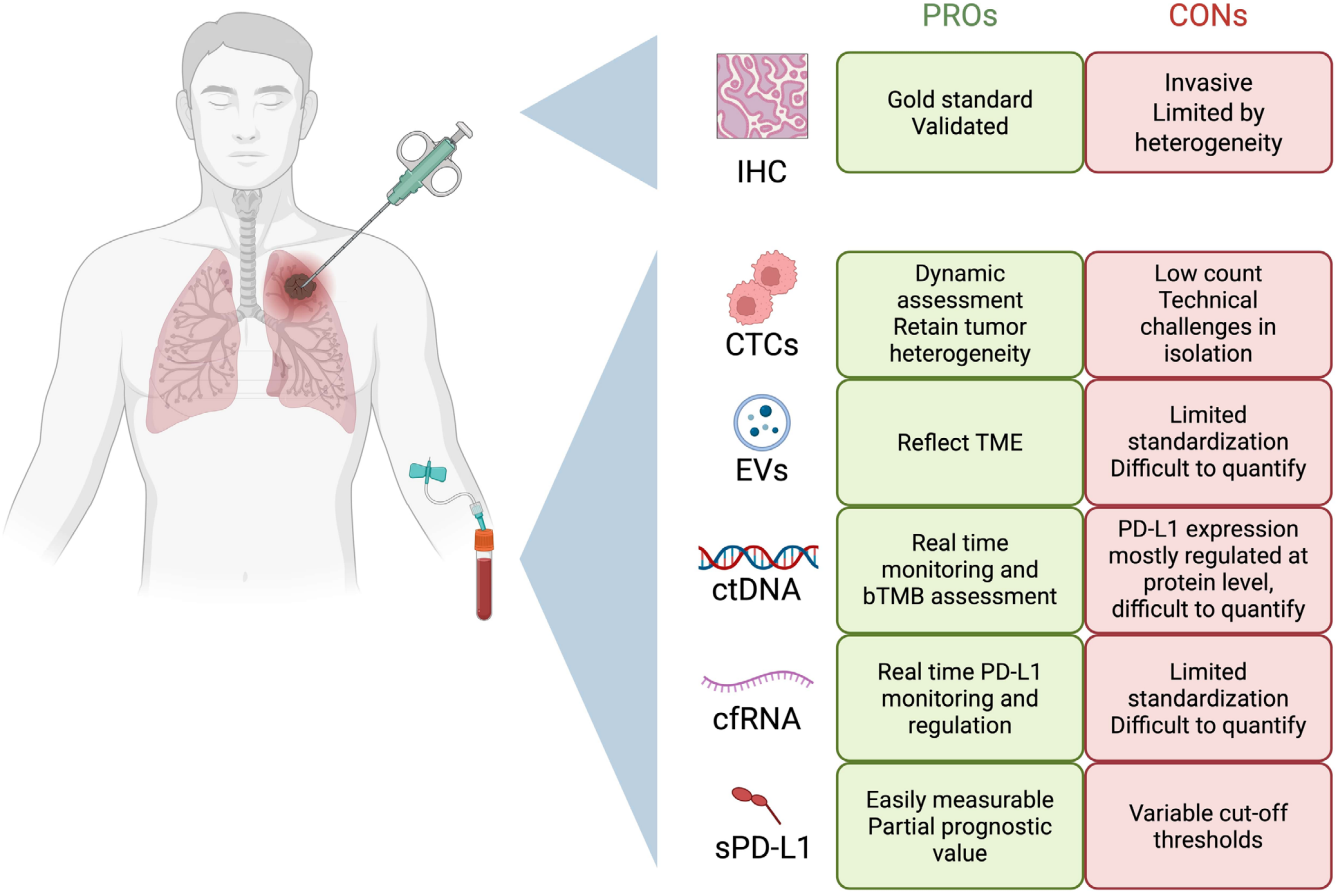
Advancing PD‐L1 Testing in NSCLC through liquid biopsy.

Liquid biopsy has transformed the field of oncology by offering a minimally invasive approach to detect tumour‐derived biomarkers in body fluids, with promising applications in cancer screening, early diagnosis, treatment monitoring, and prognostication. It comprises various circulating biomarkers, including CTCs, EVs, ctDNA, cfRNA, and soluble proteins, which can be found in multiple body fluids, such as blood, urine, cerebrospinal fluid, or saliva [23, 30, 31]. Each of these biomarkers provides distinct biological insights into tumour progression, therapeutic response, and resistance mechanisms [30, 31]. Compared with tissue biopsies, liquid biopsies offer the advantages of minimal invasiveness, repeatability, and the potential to reflect spatial and temporal heterogeneity. In oncogene‐driven NSCLC, liquid biopsy has already transformed clinical practice by enabling real‐time detection of actionable mutations and resistance mechanisms [23]. Based on the promising results obtained with the use of liquid biopsy to guide treatments in oncogene addicted NSCLC, there is an increasing effort to bring it into the domain of immunotherapy, where it offers the potential for rapid assessment of predictive biomarkers at baseline or during treatment. Extending this approach to immunotherapy‐related biomarkers, including PD‐L1, represents an active area of investigation rather than an established clinical paradigm (Figure 1).

### 4.1 PD‐L1 Assessment Using Liquid Biopsies

#### 4.1.1 Circulating Tumour Cells

CTCs are cells that are shed from the primary tumour into the bloodstream, playing a crucial role in metastasis, and their presence in NSCLC has been correlated with tumour burden, disease stage, and overall survival [32]. Unlike other circulating biomarkers, CTCs provide intact cellular material, allowing phenotypic and functionalcharacterisation, including PD‐L1 expression [33]. However, their rarity, technical complexity, and lack of standardised isolation methods limit routine clinical use. Current approaches include EpCAM‐dependent platforms, such as CellSearch, the only FDA‐approved CTC assay for prognostic use in selected cancers, and EpCAM‐independent methods that rely on physical or biological properties, which are particularly useful in the context of epithelial‐to‐mesenchymal transition (EMT) [34, 35, 36].

Studies evaluating PD‐L1 expression on CTCs in NSCLC have reported heterogeneous results, with concordance rates with tissue PD‐L1 ranging from low to high across different cohorts [37, 38, 39, 40, 41, 42, 43, 44]. Importantly, PD‐L1 expression on CTCs appears to be dynamic and may provide real‐time information on treatment response. Several studies suggest that decreasing PD‐L1 expression on CTCs during immunotherapy is associated with clinical benefit, whereas persistent or increasing expression correlates with resistance, although results remain inconsistent and largely derived from small, exploratory cohorts [43, 44, 45] (Table 2). Rather than serving as direct surrogates for tissue PD‐L1, CTCs may offer unique insights into tumour evolution, immune escape, and treatment resistance. The strong association between PD‐L1 expression, EMT, and proliferative or stem‐like CTC phenotypes highlights their biological relevance but also underscores the need for cautious interpretation.

**TABLE 2:** Clinical studies evaluating PD‐L1 assessment through liquid biopsy approaches in NSCLC.

Reference	Study population	Sample size	Liquid biopsy type	Detection method	PD‐L1 cut‐off	Main findings
Dall'Olio, 2021^39^	Advanced NSCLC on ICIs (2nd/3rd line)	39	CTC	CellSearch	≥ 1 PD‐L1+ CTC	No correlation between PD‐L1 expression on CTCs and matched tumour tissue
Zhou, 2023^38^	Advanced NSCLC	139	CTC	IF (Cyttel)	Semi‐quantitative staining scale	Higher PD‐L1 detection on CTCs vs. tissue; PD‐L1 positivity associated with longer PFS
Ilie, 2018^41^	Advanced NSCLC	106	CTC	ISET + IHC (SP142)	≥ 1 PD‐L1+ CTC	High concordance between PD‐L1 on CTCs and tissue; worse PFS in PD‐L1+ CTC patients treated with chemotherapy
Cheng, 2020^44^	Stage II‐IV NSCLC	59	CTC	IHC	≥ 1 PD‐L1+ CTC	80% concordance between CTC and tissue PD‐L1; PD‐L1+ associated with shorter PFS
Janning, 2019^37^	Advanced NSCLC	127	CTC	CellSearch + Parsortix	≥ 1 PD‐L1+ CTC	Increase in PD‐L1+ CTCs during immunotherapy associated with progression; no tissue correlation
Sinoquet, 2021^40^	Advanced NSCLC	54	CTC	CellSearch	≥ 1 PD‐L1+ CTC	54% concordance with tissue PD‐L1; PD‐L1+ CTCs associated with poor prognosis
Guibert, 2018^42^	Advanced NSCLC on ICIs (2nd line)	96	CTC	ISET + Western blot	≥ 1 PD‐L1+ CTC	PD‐L1+ CTCs enriched in non‐responders; no correlation with tissue PD‐L1
Spiliotaki, 2023^45^	Advanced NSCLC on ICIs (1st line)	47	CTC	IF + Ki‐67	Intensity‐based	PD‐L1+ CTCs + Ki‐67 > 30% correlated with shorter PFS/OS.
Ikeda, 2021^43^	Advanced NSCLC	45	CTC	MCA system	PD‐L1 ≥ 7.7%	Early decline of PD‐L1+ CTCs associated with better response
Akbar, 2023^51^	Advanced NSCLC	17	EV	Ultracentrifugation + ELISA	exoPD‐L1 > 20.36 pg/mL	Increase in exosomal PD‐L1 observed in non‐responders
De Miguel Perez, 2022^53^	Advanced NSCLC	43	EV	Ultracentrifugation + immunoaffinity	PD‐L1 + EVs > 9.5 pg/mL	Exosomal PD‐L1 associated with immune suppression and reduced ICI efficacy
van der Leest, 2021^58^	Advanced NSCLC	104	ctDNA	NGS	Not specified	Early ctDNA reduction predicted survival; no correlation with tissue PD‐L1
Anagnostou, 2023^59^	Advanced NSCLC	102	ctDNA	NGS	NA	ctDNA clearance associated with improved OS independently of tissue PD‐L1
Ricciuti, 2021^60^	Advanced NSCLC	89	ctDNA	NGS	NA	ctDNA dynamics predicted ICI benefit better than PD‐L1 IHC
Sha, 2020^61^	Advanced NSCLC	120	ctDNA (TMB)	NGS	TMB‐high ≥ 10 mut/Mb	Blood TMB showed limited correlation with tissue PD‐L1
Walker, 2023^66^	Advanced NSCLC	53	cfRNA (PD‐L1 mRNA)	qPCR	≥ 1 copy/μL	Plasma PD‐L1 cfRNA predictive of ICI benefit, comparable to tissue IHC
Fernandez, 2022^67^	Advanced NSCLC	85	cfRNA (PD‐L1/2 mRNA)	qPCR	≥ 1 copy/μL	High PD‐L1 cfRNA correlated with longer OS; strong negative predictive value
Conroy, 2019^68^	Advanced NSCLC	68	cfRNA (PD‐L1 mRNA)	RNA‐seq	Not specified	PD‐L1 transcript profiling comparable to IHC in predicting ICI response
Okuma, 2017^71^	Advanced NSCLC	96	sPD‐L1	ELISA	Not specified	High plasma sPD‐L1 associated with poor prognosis

Abbreviations: cfRNA, cell‐free RNA; ctDNA, circulating tumour DNA; CTCs, circulating tumour cells; ELISA, enzyme‐linked immunosorbent assay; EV, extracellular vesicles; ICI, immune checkpoint inhibitors; IF, immunofluorescence; MCA system, microcavity array; miRNA, microRNA; NA, not applicable; NGS, next generation sequencing; NSCLC, non‐small cell lung cancer; PD‐L1, Programmed Death‐Ligand 1; qPCR, quantitative PCR; sPD‐L1, soluble PD‐L1; TMB, tumour mutational burden.

#### 4.1.2 Extracellular Vesicles

EVs are important messengers in intercellular communication, transferring information between tumour cells and immune cells. These vesicles are categorised into three main types on the basis of their size, formation and release mechanisms: Exosomes, microvesicles, and apoptotic bodies Among these, tumour‐derived exosomes play a central role in intercellular communication and immune modulation [46]. In particular, they can promote immune evasion by carrying PD‐L1 and suppressing T‐cell activation [47, 48]. Among the methods available for EV isolation andcharacterisation, traditional ultracentrifugation is the most commonly used. Other approaches include precipitation‐based methods, which are faster but prone to contamination; size‐exclusion chromatography, which typically yields cleaner samples at the cost of some vesicle loss; and emerging microfluidic technologies [47, 48].

Several studies have reported correlations between PD‐L1 levels on EVs and tumour PD‐L1 expression, as well as associations with immunotherapy response and resistance [48, 49, 50, 51, 52, 53, 54] (Table 2). In particular, PD‐L1‐positive Evs can interfere with anti‐PD‐L1 therapy by acting as decoys that sequester therapeutic antibodies, thereby preventing T‐cell activation [48]. In this way, PD‐L1‐positive Evs could serve as predictive biomarkers for resistance to ICIs. Nevertheless, methodological heterogeneity in EV isolation, quantification, and PD‐L1 detection remains a major barrier [48]. While PD‐L1‐positive Evs represent a biologically compelling biomarker, their clinical utility has not yet been established, and findings across studies remain variable. In addition, inhibiting EV‐mediated PD‐L1 secretion has emerged as a novel therapeutic strategy in preclinical models [54]. However, targeting exosome secretion should be approached with caution, as Evs play crucial roles in both physiological and pathological processes, and uncontrolled T‐cell activation could lead to immune‐related adverse events, necessitating further investigation in both preclinical and clinical settings.

#### 4.1.3 Circulating Tumour DNA

ctDNA, a subset of cfDNA, is released into the bloodstream after apoptosis or necrosis of tumour cells and provides a non‐invasive method for detecting tumour‐specific genetic alterations, including mutations, copy number variations, and methylation changes. ctDNA analysis, through Next Generation Sequencing (NGS) or digital droplet Polymerase Chain Reaction (PCR), has become an integral component of NSCLC management for genomic profiling and disease monitoring [55, 56].

Although ctDNA does not directly measure PD‐L1 protein expression, it provides indirect immunotherapy‐relevant information, including blood‐based TMB and early molecular response [57, 58, 59, 60, 61]. Serial ctDNA clearance has consistently been associated with improved outcomes in patients receiving ICIs. Efforts to link ctDNA features with PD‐L1 expression remain exploratory [62, 63]. Differences between tissue‐based and blood‐based TMB, lack of standardised cut‐offs, and platform variability currently limit clinical application for immunotherapy selection [10] (Table 2).

#### 4.1.4 Circulating Cell‐Free RNA

Different from ctDNA, which primarily reflects genetic mutations, cfRNA offers insight into active gene transcription and post‐transcriptional regulation. Among cfRNA, microRNAs (miRNAs) are small, non‐coding RNAs that can be found circulating freely or encapsulated in Evs and play a role in regulating gene expression at a post‐transcriptional level and in tumour immune evasion [64, 65]. As compared to ctDNA, miRNAs are more stable in circulation and can be detected using relatively simple assays, including NGS or quantitative reverse transcription PCR. This stability, combined with their regulatory role in tumour biology, makes miRNAs a promising biomarker for improving patient selection in immunotherapy [64, 65].

Plasma PD‐L1 mRNA levels and PD‐L1‐related miRNA signatures have shown encouraging correlations with tissue PD‐L1 expression and immunotherapy outcomes in early studies [66, 67, 68] (Table 2). Recent assays have shown that cfRNA‐based PD‐L1 measurements can predict response to ICIs, with higher PD‐L1 and PD‐L2 mRNA levels associated with improved outcomes. Conversely, low PD‐L1 mRNA levels appear to have negative predictive value. In addition, specific miRNAs involved in PD‐L1 regulation, such as miR‐138‐5p and members of the miR‐200 family, have emerged as potential biomarkers of immunotherapy response [66, 67, 68]. However, these findings require validation in larger cohorts and harmonisation of analytical methods before clinical translation.

#### 4.1.5 Soluble PD‐L1

Soluble PD‐L1 (sPD‐L1), detectable in plasma, is produced through two primary mechanisms: Alternative splicing of PD‐L1 mRNA and proteolytic cleavage of membrane‐bound PD‐L1, reflecting immune modulation [69, 70]. Enzyme‐linked immunosorbent assay (ELISA) is the most commonly used detection method.

Elevated baseline or increasing sPD‐L1 levels during treatment have been associated with poorer outcomes in several studies [69, 70, 71, 72] (Table 2). Importantly, dynamic changes in sPD‐L1 levels appear more informative than baseline values alone [69, 70, 71, 72]. While technically accessible, variability in assay platforms and cut‐off definitions has limited its adoption as a reliable predictive biomarker.

### 4.2 Challenges and Limitations

Although liquid biopsy approaches offer exciting research opportunities, they currently lack the technicalstandardisation, analytical robustness, and clinical validation required for routine PD‐L1 assessment. One key challenge is the low abundance of circulating tumour components. CTCs, for example, are rare, which makes their detection highly dependent on very sensitive technologies. Current methods often lack sensitivity and reproducibility, especially in patients with low tumour burden [35, 36]. Similar issues affect Evs, where a lack of standardised protocols for isolation, quantification, and PD‐L1 measurement continues to limit clinical adoption [46]. ctDNA poses its own difficulties, as it is often present at very low levels compared with background cfDNA, particularly in early‐stage disease. Although ultra‐deep sequencing has improved sensitivity, it remains expensive and computationally demanding, restricting its use in everyday practice [73]. For sPD‐L1, improved enzyme‐linked immunosorbent assay (ELISA) assays have increased analytical accuracy, but further harmonisation is still needed to define clinically meaningful cut‐off values and ensure consistency across studies [69]. Beyond technical limitations, most available studies are small, heterogeneous, and exploratory, often using distinct platforms and endpoints [74, 75, 76]. As such, liquid biopsy biomarkers should be viewed as hypothesis‐generating tools rather than established alternatives to tissue IHC (Figure 1).

## 5 Future Directions

The rapid evolution of liquid biopsy technologies is opening new avenues for understanding tumour‐immune interactions in NSCLC. At present, most advances remain firmly within the research domain, yet they provide valuable insights that may ultimately inform clinical decision‐making.

### 5.1 Research Opportunities

Emerging single‐cell and multi‐omics approaches are reshaping our understanding of tumour heterogeneity and immune escape. In the context of CTCs, single‐cell transcriptomic and proteomic profiling has revealed subpopulations with distinct immune‐evasive phenotypes, including EMT‐associated and stem‐like states linked to resistance to ICIs [41]. These findings highlight the potential of liquid biopsy not only as a biomarker platform but also as a window into resistance biology. Similarly, ctDNA analysis is moving beyond mutation detection. Novel techniques such as fragmentomics, methylation profiling, and nucleosome footprinting allow increasingly refined characterisation of tumour dynamics and immune responsiveness [19, 77, 78]. These approaches may prove particularly valuable for early identification of non‐responders to immunotherapy and for monitoring molecular response well before radiographic progression becomes evident. Parallel to these technological developments, computational and artificial intelligence (AI)‐based tools are increasingly being applied to integrate complex datasets derived from liquid biopsy, tissue pathology, imaging, and clinical parameters. Early studies suggest that AI‐driven models may outperform single biomarkers in predicting immunotherapy benefit, supporting a shift toward composite, multidimensional predictors rather than reliance on PD‐L1 expression alone [79].

### 5.2 Clinical Implementation Pathways

From a clinical perspective, rather than replacing tissue IHC, liquid biopsy‐based PD‐L1 assessment is best viewed as a complementary strategy that may add clinically relevant information in selected cases where tissue‐based evaluation is limited or potentially misleading. Indeed, increasing evidence suggests that blood‐based biomarkers may capture clinically relevant aspects of tumour biology that are not always reflected by a single tissue sample [23, 30, 76]. Liquid biopsy could be particularly valuable when tumour tissue is limited or inaccessible. In advanced NSCLC, diagnostic samples are frequently small and obtained through minimally invasive procedures, often leaving limited material after histopathologic and molecular characterisation [20, 74]. This issue is particularly relevant for PD‐L1 testing, where biopsy size and sampling site may significantly influence the reliability of results [20, 74]. In this setting, liquid biopsy‐based PD‐L1 assessment may provide an additional source of biologically informative material.

Liquid biopsy approaches may also help address the well‐recognised issue of spatial and temporal heterogeneity. Multiple studies have demonstrated discordance in PD‐L1 expression between primarytumours, metastatic lesions, and small biopsy samples [18, 21, 41]. Clinically, this may partly explain why some patients with low or negative tissue PD‐L1 expression still derive benefit from ICIs, whereas others with high PD‐L1 tumours experience primary resistance. In this context, serial blood‐based monitoring could offer a more dynamic representation of tumour‐immune interactions over time compared with a single baseline tissue biopsy.

In addition, dynamic changes in blood‐derived immune biomarkers during treatment have been associated with treatment response, survival outcomes, and emerging resistance mechanisms. In particular, longitudinal monitoring strategies may allow earlier identification of non‐responders compared with conventional radiologic assessment, potentially supporting more adaptive therapeutic approaches [59, 60, 62].

Overall, although these approaches remain investigational and are not currently recommended by international guidelines for routine clinical decision‐making, the growing body of evidence supports their potential role as complementary tools in selected clinical settings. Their future integration in immunotherapy decision‐making will require prospective validation, assayharmonisation, and the establishment of clinically meaningful and reproducible thresholds across different platforms and patient populations.

## 6 Conclusions

The integration of liquid biopsy technologies into the evaluation of PD‐L1 expression in lung cancer represents a significant development in precision oncology. Our review highlights the promise of circulating biomarkers as valuable companions to traditional tissue assessments, providing real‐time insights into immune activity and treatment responses. As we continue to explore the complexities of cancer immunotherapy, these innovative diagnostic tools not only hold the potential to refine therapeutic strategies but also enhance patient selection for ICIs. However, the journey toward routine clinical adoption is fraught with challenges, including the need forstandardisation, rigorous validation, and improved sensitivity and specificity of these assays. Future research must prioritise unlocking the full potential of liquid biopsies by employing advanced technologies, such as multi‐omics approaches and AI, to enhance predictive accuracy and clinical relevance. At present, liquid biopsy approaches should be regarded as investigational tools with strong biological rationale but limited clinical readiness. Continued methodological refinement, standardisation, and prospective validation will be critical to determine whether these technologies can meaningfully enhance immunotherapy selection and outcomes in NSCLC.

## Author Contributions

Conceptualisation, R.L., V.C., A.A., and G.G.; Writing – original draft preparation, R.L., V.C., and A.A.; Writing – review and editing, R.L., V.C., A.A., B.R., E.C., L.T., K.D.E., C.C., A.E., I.M., V.Ca., and S.B.; Supervision, M.D., F.B., and G.G.; Methodology, A.E., C.C., I.M., V.Ca., and S.B.; Visualisation, R.L. and B.R., Project administration, R.L. and M.P. All authors have read and agreed to the published version of the manuscript.

## Funding

This study was supported by open access funding provided by the University of Modena and Reggio Emilia. Preparation of this manuscript was partly supported by funding from the European Union—NextGenerationEU through the Italian Ministry of University and Research under PNRR—M4C2‐I1.3 Project PE_00000019 “HEAL ITALIA” to Chiara Chiavelli, CUP UNIMORE E93C22001860006.

## Conflicts of Interest

The authors declare no conflicts of interest.

## Data Availability

Data sharing not applicable to this article as no datasets were generated or analysed during the current study.
